# Long-Term Stability of Visual Pattern Selective Responses of Monkey Temporal Lobe Neurons

**DOI:** 10.1371/journal.pone.0008222

**Published:** 2009-12-09

**Authors:** Igor V. Bondar, David A. Leopold, Barry J. Richmond, Jonathan D. Victor, Nikos K. Logothetis

**Affiliations:** Max Planck Institut für Biologische Kybernetik, Tübingen, Germany; Mount Sinai School of Medicine, United States of America

## Abstract

Many neurons in primate inferotemporal (IT) cortex respond selectively to complex, often meaningful, stimuli such as faces and objects. An important unanswered question is whether such response selectivity, which is thought to arise from experience-dependent plasticity, is maintained from day to day, or whether the roles of individual cells are continually reassigned based on the diet of natural vision. We addressed this question using microwire electrodes that were chronically implanted in the temporal lobe of two monkeys, often allowing us to monitor activity of individual neurons across days. We found that neurons maintained their selectivity in both response magnitude and patterns of spike timing across a large set of visual images throughout periods of stable signal isolation from the same cell that sometimes exceeded two weeks. These results indicate that stimulus-selectivity of responses in IT is stable across days and weeks of visual experience.

## Introduction

A central idea in sensory physiology is that perception arises as a consequence of the neuronal activity patterns in the brain elicited by stimuli and objects in the external world. Inferotemporal cortex of monkeys is thought to be critical in forming these perceptions because ablations of area TE severely impair visual recognition [1], and lesions in the superior temporal sulcus (STS), including the fundus, adversely affect visual learning [2]. Supporting the interpretation that inferotemporal cortex is critical for these perceptions, single neurons in these temporal lobe brain regions respond selectively to complex and often meaningful visual stimuli [3]–[7]. Neurons in area TE are selective for particular stimulus patterns [8], [9], they seem related to perception [10]–[12], and they can respond to stimuli categorically [13]–[15]. The selectivity of neuronal responses in TE seems to be shaped by visual experience over both short and long time scales [14], [16]–[21]. Selective responses are also seen in neurons in the superior temporal sulcus (STS) [3], [22]–[24], and understanding the differences between the two brain regions is of great interest [25], [26].

Given the inherent plasticity in these areas, it is possible that daily experience continually reshuffles how individual neurons respond to shapes and objects. On the other hand, it may be that visual recognition depends on preserving individual IT neurons' previously established selectivity, in which case their response characteristics might, by default, remain the same day after day. Despite the importance of this information for interpreting inferotemporal lobe function, there are few investigations addressing this issue, almost certainly because of the difficulty in monitoring the activity of a single neuron across days in monkey cortex. Recently reports of long-term recordings from monkeys have begun to appear [27]–[29], indicating that, despite the remaining difficulties, the technical obstacles are being overcome. In the present study we monitored isolated neurons in the inferotemporal cortex of two rhesus monkeys using arrays of up to 64 microwires that were chronically implanted. We were able to record signals from single neurons over periods lasting up to 17 days. We found that neurons in both TE and STS maintained selectivity in both their firing rates and spike timing patterns across our large stimulus set for up to a couple of weeks.

## Results

While two rhesus fixated a small spot sixty-five images selected from photographs and drawings of birds, mammals, insects, vegetables, and human faces and hands were shown repeatedly behind the spot, in pseudorandom order each day (**Figure S1**, see **Methods**). The images were shown for 400 ms. Each image appeared between 10 and 68 times in a recording session. The images had no known particular behavioral significance. The monkeys were familiar with both the task and the stimuli prior to the neurophysiological recordings.

The monkeys were implanted with chronic electrode bundles in two inferior temporal subregions, TE near the anterior medial temporal sulcus (AMTS, monkey N97) and area PGa in the superior temporal sulcus (STS, monkey E98) [30] (details in **Methods**, Supplementary Methods S1 and **Figures S2** and **S3**). The electrode bundles containing 64 high impedance microwires were surgically implanted so that the tips were in the target location. A microdrive for advancing their position, and a 64-channel connector attached to a chamber on the animal's head. Each day, all channels were evaluated for the presence of action potential waveforms, to determine which electrodes were monitoring well-isolated single neurons. Up to 12 channels showed prominent action potentials on a given day, with one or two units present in the signal from each electrode, although on occasion as many as 4 units were distinguishable (**Figure S4**). Every few weeks, the array was advanced approximately 100 µm by turning a small screw on the manual microdrive (see **Figure S3**) to sample a new population of neurons. This intervention generally resulted in the isolation of new units, although sometimes the procedure had to be repeated on a couple of successive days to do so. Recordings were carried out over a period exceeding one year in the case of monkey N97 and 2.5 months in case of monkey E98, with test sessions separated by intervals ranging from a day to several weeks. Data were collected during 53 recording sessions in monkey N97, and 20 sessions in monkey E98 (see **Figure S5**). During this period, the monkeys' only behavioral task was fixation during the repeated presentation of the same sixty-five stimuli, presented in pseudo-random order, for 1–2 hours each day. Outside of the testing sessions their daily visual experience was typical of primates in a group-housed environment. They interacted with conspecifics, received food and treats from the investigators and animal caretakers, and manipulated toys and various other objects in the cage.

### Neurons in IT maintain their selectivity from day to day

**Figure 1:**
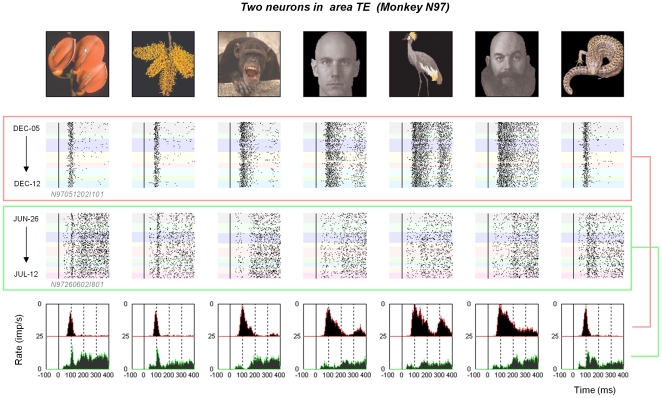
Single unit responses in area TE. Data are shown from two neurons from monkey N97. The two neurons were recorded on two different microwires (channels of electrodes bundle marked as I1 and I8) and during two different time periods. Directly below each image, the action potential responses are shown over a period of several days, with each background color corresponding to data collected from a different session. The diverse responses appear to be stable over the recording periods. At the bottom are the corresponding peristimulus time histograms for the two neurons.

**Figure 2:**
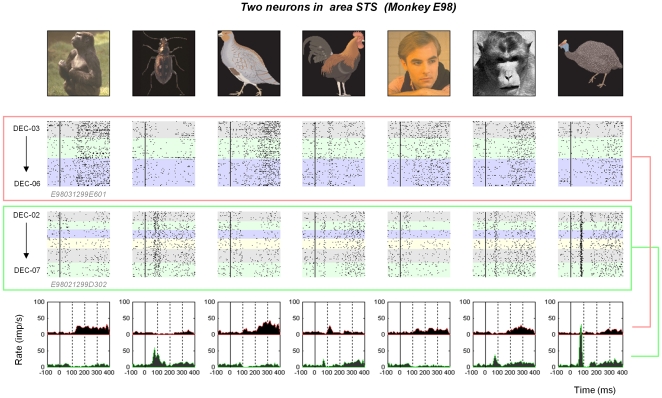
Single unit responses in the STS. Data are shown from two neurons collected on two different microelectrodes from monkey E98 in the same format as **Figure 1**. The data were collected simultaneously for three days, after which the lower neuron was monitored for an additional 3 days.

In total, we identified 192 different neurons, with 159 (83%) showing visual responses to at least one of the stimuli in the set (93 in N97 and 66 in E98) with 69 of these seemingly stably isolated over at least two consecutive recordings sessions. In a few cases, we monitored neurons for over two weeks, though the majority of neurons were present for a week or less (the distribution of neuron isolation durations for each monkey is shown in **Figure S8**). Single units in TE and STS responded reliably to stimuli in the test set, with responses appearing to show the same stimulus selectivity in both spike count and temporal response patterns [31] across the days of isolated neuronal recording (**Figures 1** and **2**; responses to all stimuli for one cell #*N97260602I80*, are shown in **Figure S6**).

**Figure 3:**
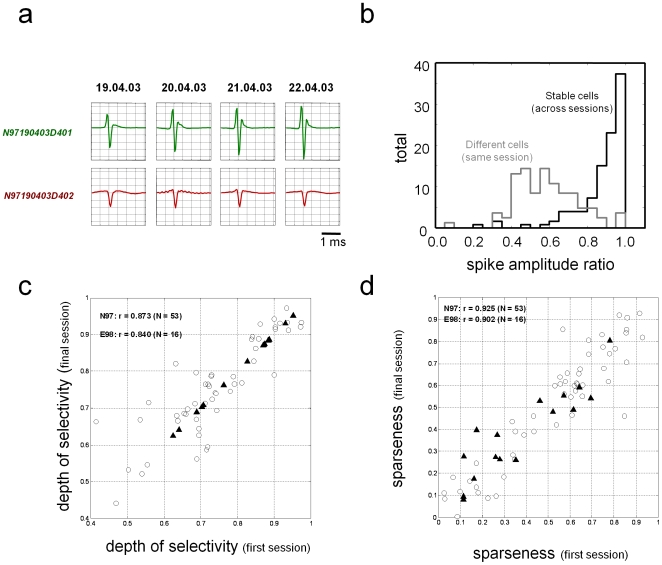
Waveforms remained similar between sessions. (a) The waveforms of the simultaneously recorded neurons shown in **Figure 5** over the stable recording period. The basic shape remained similar, though the spike amplitude drifted over time. (b) Comparison in the ratio of the spike waveform amplitudes across the population (see **Methods**). Stable neurons recorded across multiple sessions had amplitude ratios near 1.0, indicating that they did not change much over time. The amplitude ratio between different neurons recorded from the same session was generally much lower. (c) Correlation (r) in the depth of selectivity between the first and final sessions of all stably recorded neurons. Data shown separately for monkey N97 (open circles) and monkey E98 (black triangles) (d) Correlation (r) in the sparseness index between the first and final sessions of all stably recorded neurons.

We used two independent approaches to evaluate stability across sessions. First we compared the waveform shape and amplitude, interspike interval distribution, and other spiking statistics across successive recordings sessions (**Figure 3a and b**, **Figure S5**). Then, for multi-session epochs in which these parameters were stable, we evaluated the stability of the stimulus selectivity by computing two standard indices, depth of selectivity [32] and sparseness[33], based on the spike rates across stimuli (see Supplementary Methods S1). Although these measures had broad ranges of values across the 69 neurons, the values were similar across first and last sessions for each neuron (**Figure 3c and 3d**, also **Figure S7**), indicating that the basic selectivity profile did not change across sessions and was similar between cortical areas PGa and AMTS.

**Figure 4:**
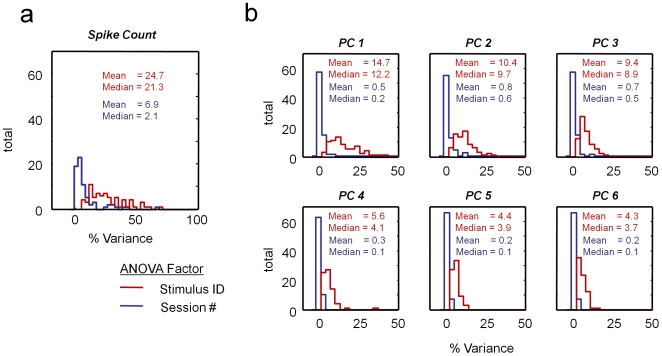
Comparison of variance attributed to stimulus selectivity vs. session number across the population of stably recorded neurons (N = 72). A 2-way ANOVA was used to compute the percent variance attributed to these two factors. (a) Distribution of variance in the spike count (40 to 440 ms following stimulus onset) over stimulus and session. (b) Same analysis as (a), but for the variance in the coefficients of the first six principal components. Principal components were computed for each neuron individually based on the mean responses to the fifteen stimuli eliciting the highest responses. For each neuron, the coefficients corresponding to each trial were then computed as the inner product of the individual trial histogram with the first six principal components (see **Methods**).

To compare the strength of neural response selectivity across different stimuli to day-to-day variation in spiking responses to the same stimuli, we used a 2 way ANOVA to calculate the variance accounted for by two main factors, *stimulus identity* and *session number*, for the 72 neurons recorded across two or more days. Given the prominent temporal patterning in the spike timing (cf **Figures 1** and **2**), we also examined the response time course using principal component analysis (PCA). For each neuron, the principal components were computed from the average responses to the fifteen stimuli eliciting the strongest responses (see **Methods** for details). The ANOVA analysis was then applied to the spike counts and each of the first six PCA coefficients for each cell for all trials. The ANOVA responses for each of the examples presented in the paper are shown in **Table S1**. A substantial and significant proportion of the variance is explained by stimulus identity (**Figure 4**, red histograms), that is, the responses are stimulus selective [34]. Only a very small percentage of the variance can be attributed to changes across sessions (**Figure 4**, blue histograms). The ANOVA did reveal small but significant session-to-session variability in the overall spike count for some cells (e.g. lower example in Figure 2), but the *session* factor generally contributed less than 1% of the variance in the spike count for each of the first three principal components. Thus, as inspection of the rasters suggests, the complex stimulus selectivity of IT neurons is stable over days, and using the criteria applied here, there was very little contamination due to recording problems arising across days.

### Comparison with selectivity of adjacent neurons

**Figure 5:**
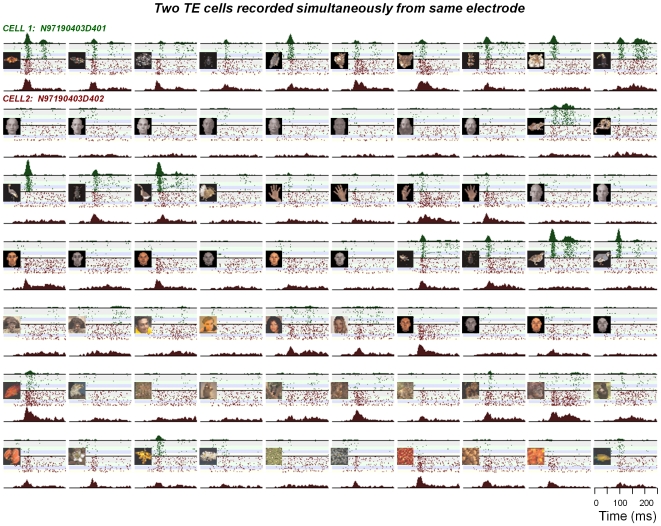
Responses of two area TE neurons to 65 stimuli. The two neurons were recorded simultaneously from the same electrode over a period of four days. The stimuli, presented at time = 0, are shown in the left portion of each panel. Despite the proximity of the two cells, their responses, including their basic stimulus selectivity, remained distinct over the recording sessions. Accumulated peristimulus time histograms are shown above and below the rasters of the two neurons.

**Figure 6:**
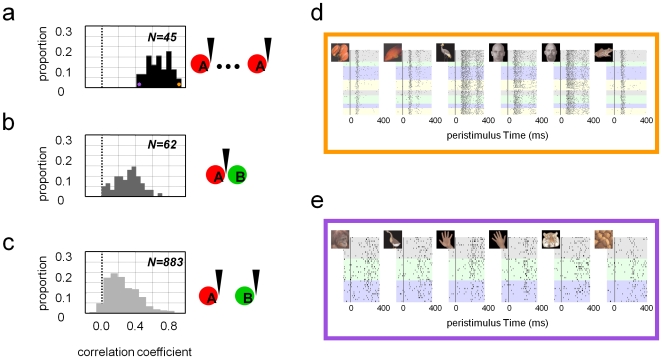
Evaluation of stability over the population of recorded neurons. A correlation-based similarity index (CSI) was used to characterize the similarity of the selective responses between the same neuron measured in the first and last recording sessions from the same electrode (a), between different neurons measured simultaneously on the same electrode (b), and between different neurons measured simultaneously on different electrodes (c). In the last case, only neurons for which spike waveforms were successfully maintained over at least 2 sessions were considered. Panels (d) and (e) show two neurons with the highest and lowest CSI between sessions, corresponding to the orange and purple dots in (a), respectively.

The stability of the recorded spike shape, combined with maintained selectivity in spike count and temporal patterning, provided strong evidence that individual neurons were successfully monitored for up to a fortnight. However, there are a couple of lingering issues to address. One is that different neurons might have similar spike waveforms and ISI distributions. Also, neighboring neurons in the inferotemporal cortex seem to cluster in that they are responsive to a largely overlapping subset of stimuli [7]. We therefore carefully examined the possibility that the maintained selectivity observed between sessions might, in fact, arise from successive isolations of *different* neurons with almost identical spike waveform and response selectivity profiles. We exploited the fact that the microwire bundle provided many instances of stable recordings of more than one neuron from the same electrode, making it possible to analyze the responses of pairs of neurons that were recorded simultaneously on single electrodes, i.e., they ought to be adjacent or at least physically close. In general, the preferences of adjacent neurons in our sample were different in detail. In the example shown in **Figure 5**, for instance, the responses of two such TE neurons (green and red) are shown for each of the images in the stimulus set (**Figure S1**). Both neurons were recorded for four successive days, with the mean spike waveforms for each day shown in **Figure 3a**. The rasters show that the response selectivity for each neuron was stable over days, while the two neurons differed markedly in their stimulus selectivity. A three-way ANOVA of the spike count revealed a significant neuron vs. stimulus interaction (F = 33.25, df = 69, p<0.0001), accounting for 8.2% of the variance. In this example, the interaction between session vs. stimulus was also significant (F = 1.79, df = 207, p<0.0001), suggesting some day to day drift in responses; however, these changes accounted for less than 2% of the total variance for this neuron. The same analysis applied to the first principal component data revealed a significant interaction only in the neuron vs. stimulus interaction (F = 29.56,df = 69,p<0.0001), but not the session vs. stimulus interaction (F = 1.11,df = 207,p = .14), showing that the session to session variance had little influence on the aspects of the signal related to the stimulus selectivity. To analyze this across the 45 neuron pairs from single electrodes, we applied a correlation-based similarity index (CSI) that simultaneously accounts for the magnitude and temporal response to each of the 65 stimuli being presented (see **Methods**). A CSI of 1.0 would occur if both the magnitude and temporal dynamics of the response to each stimulus were identical for two conditions under comparison. The CSI was much higher for the putative stably isolated neurons over multiple days (**Figure 6** median 0.67, n = 45) than for neurons collected from different neurons between sessions (median 0.18, n = 883). In addition, neurons collected on the same day from the same electrode also exhibited a CSI that was markedly lower than the stably isolated neurons (median = 0.31, n = 62). Several factors can affect the CSI of even a perfectly isolated neuron collected at two different times. These include (1) the inherently high response variability of cortical neurons, (2) either very high or very low selectivity levels (intermediate selectivity provides the most robust CSI), and (3) low spiking rate. The higher correlations in **Figure 6a**, when considered in light of the lower correlations in **Figure 6b **and **c** make it unlikely that different neurons were recorded on subsequent sessions. Examples of neurons displaying the highest and lowest CSI values across days are shown in **Figure 6d **and **e**.

## Discussion

Chronic monitoring of single cortical neurons is desirable for investigating how external sensory stimuli are represented in the brain. The inferotemporal cortex is of particular interest in this regard, since the responses of neurons there are known to be stimulus-specific [5], [6], malleable [14], [16]–[21] and closely linked to perception [10]–[12]. In the present study, we demonstrated two new points. First, it is possible to monitor activity of individual inferotemporal neurons with microwire electrodes, and to track their selectivity over sessions, sometimes up to two weeks. Second, for the single inferotemporal neurons recorded here, complex selectivity remains stable over these periods.

It has been possible for some time now to record neurons over periods of days or weeks in several species [28]. It would seem that a straightforward way to ensure that successive recordings reflect activity of the same neuron is to show that the spike waveform is constant across recording sessions. But when this criterion is applied to primate recordings [27], [29], spikes waveforms measured from a single cell may change their shape and amplitude over time–and spikes measured from two different cells often appear identical. For these reasons, it is sometimes deemed necessary to use technical advances, such as multicontact “tetrodes” to better isolate and identify individual cells from session to session [29]. In contrast, the present study identified putative stable isolations based on parameters of the spike waveforms measured on single microwires. The similarity of several parameters, including waveform shapes, amplitudes, ISI histograms, and channels of origin, across days makes it unlikely that more than a negligible fraction of such neurons were misclassified from day to day. Nonetheless, it is important to understand the consequences of occasionally misclassifying a neuron. The errors fall generally into two categories: (1) failure to identify a neuron as being the same as before (i.e. “false negative”), and (2) classifying a new neuron as the same in a previous session (i.e. “false positive”). Neither of these errors would lead to the observation that neurons maintained their selectivity over extended periods of time. In the case of the false negative error, a single neuron would be treated as two distinct ones with shorter periods of stable isolations. Although this may inflate the sample size, it would not lead to extended periods of maintained selectivity.

A more serious concern is about what effect false positives, where two inferotemporal neurons with similar waveforms might be wrongly classified as the same, would have on the results. The experiments showed that the selectivity of most neurons was virtually unchanged from day to day, with regard to (1) the general tuning parameters such as sparseness and depth of selectivity, (2) the fraction of day-to-day variance in the spike count and temporal response profile (ANOVA), and (3) the intersession correlation of response profiles to the stimulus set (CSI). But would this not also be the case with unstable isolations if the tuning of adjacent neurons is nearly identical, as might be expected given the columnar nature of responses in the visual cortex [35], including the inferotemporal cortex [7]? From the literature, this does not appear to be the case. Whereas neighboring neurons show similarity in their response preferences, they differ in the details of their selectivity. In fact, the response tuning profiles of adjacent neurons are largely independent in the primary visual cortex [36]–[38], as well as the inferotemporal cortex [34]. Our recordings of simultaneously recorded inferotemporal neurons either on one electrode or across electrodes had response tuning profiles that differed substantially in their details, much more so than those neurons stably monitored over many days (cf. **Figures 5** and **6**). In this study substantial *changes* in selectivity would have been difficult to interpret, since they could have arisen from either monitoring of new neurons, or the shifting selectivity of the same neuron. However, the stable selectivity profiles we observed could only persist if monitored the same neurons over days.

The constancy of spike timing is a particularly intriguing physiological feature of the responses that were preserved from session to session. Response patterns showed transient peaks, inhibitory valleys, oscillatory events and epochs of sustained firing, sometimes within the response to the same stimulus, and certainly across different stimuli for the same neuron. This diversity of temporal modulation has been described before in studies that have raised important questions about the nature of the neuronal code [39], [40]. For example, it seems that early and late response phases of IT responses encode different aspects of a stimulus [10], [41], [42]. We found that both early responses, which were often transient and non-selective, and late responses, which were sometimes oscillatory or inhibitory and stimulus-specific, were maintained from day to day. It should be mentioned that, while the method our study was able to highlight the high degree of day to day stability, it was not sensitive to very subtle changes (i.e. changes smaller than the response differences elicited by different stimuli), and we therefore cannot rule out that some aspects of the neurons' response properties drifted during the monitoring period. Given that responses in the inferotemporal cortex can be reinforced or diminished according to learning within a recording session [21], [43], [44], or over longer periods of time [11], [14], [17], [19], [20], future work aims to establish how the selectivity of individual neurons is shaped over time, such as during perceptual learning or the acquisition of visual expertise for a novel stimulus.

## Methods

### Subjects and behavioral task

The experiments were conducted in accordance with the guidelines set by the local authorities in Tübingen, Germany (Regierungspraesidium), and were in full compliance with the guidelines of the European Community for the care and use of laboratory animals (EUVD 86/609/EEC). Experiments were carried out on two male adult rhesus monkeys (monkey N97 and E98). Structural MRI scans confirmed that in N97 the electrode bundle was located in anterior inferotemporal cortex (AIT), and that in E98 the electrode bundle was in the fundus of the superior temporal sulcus (STS), most likely in area PGa (see Figure S3).

Each monkey was trained to perform a simple fixation task, with eye position monitored by a scleral search coil [45]. Trials started with the appearance of a fixation spot on the computer monitor, and the monkey was required to fixate his gaze on this spot throughout a trial, which consisted of two stimulus presentations. After 700–1000 ms, the first visual stimulus was presented for 400 ms (except for the first several experiments, in which a 500 ms presentation time was used), and then after a pause of 600 ms (interstimulus interval) a second stimulus was presented for the same duration. Fixation of gaze during the entire trial period was rewarded by drop of apple juice. The next trial started 3000 ms following reward (intertrial interval). Stimuli consisted of a diverse set of images, including faces, animals, objects, and geometrical patterns (see **Figure S1**). If the monkey maintained fixation during the whole trial (<3 s) within a window of 1 degree radius, a drop of apple juice was delivered as a reward. On average, each monkey performed about 600 trials per day, corresponding to the presentation of 1200 stimuli (range 350 to 3000 trials).

### Surgical procedures

All surgeries were performed under balanced general anesthesia, using aseptic techniques. Each monkey underwent two surgical procedures. In the first, a head post and scleral eye coil were implanted. In the second surgery, following several months of training, the multielectrode bundle was implanted according to coordinates identified in structural MRI scanning (see Supplementary Methods S1). In both cases anesthesia was inducted by injection of ketamine-xylazine mixture (ketamine: 10 mg/kg body weight; xylazine: 1–2 mg/kg). A catheter was introduced in the saphenous vein, and the animal was intubated in the trachea and switched to the gas anesthesia. The anesthesia regimen consisted of isoflurane 1.3% and fentanil 3 µg/kg i.v. injections, with 1.8 l/min N_2_O and 0.8 l/min O_2_. Monitoring of anesthesia state was done by the registration of CO_2_ levels, EKG, SpO_2_, temperature and blood pressure. Great care was taken to minimize perioperative pain and suffering of the animals, including housing in a warm ICU cage and daily application the analgesic Finadyne (1.0 mg/kg) for two days after surgery.

### Chronic electrodes

Electrode bundles containing 64 high impedance microwires were initially implanted 5–7 mm dorsal to the area of interest, and could be advanced by a manual microdrive (**Figure S3**, see Supplementary Methods S1). The initial lowering of the electrodes to the target was guided by search for visual responses of neurons during a later recording session when the animal was awake and presented with visual stimuli. Each microwire consisted of a nickel-chromium-aluminum core that was 12.5 µm diameter, insulated with polyimide it by the manufacturer (wire ‘IsaOhm’, Isabellenhuete, Germany). After being cut to length (110 mm), the microwires were individually attached to an 8×8 microconnector (size 7×8 mm; consisting of eight glued 8-ch microconnectors purchased from Binder, Neckarsulm, Germany) with silver-tin (‘Number 157’, Castolin, Lausanne, Switzerland). The ends of the wires were cut at an angle so that they formed a primitive kind of tip, with the lengths of individual electrodes varying over a range of 1 mm. Electrode impedance measured in saline at 1 kHz was generally 1.0–2.0 MΩ (see Ref [27]). Chronically implanted recording arrays have the advantage of permanent placement, without the need for reintroduction of the electrodes during each session, and consequently short setup times for daily recording sessions.

### Isolation of single neurons

The recordings proceeded for over two months in one animal (E98), and nearly a year in the other (N97) (see **Figure S4**). During this time, the isolations were marked by phases of exceptional stability, and others phases of relative instability. Because of the incorporation of a drive mechanism in the electrode array apparatus used for one monkey, we were able to advance the electrodes by ∼100 microns when an unstable period occurred, after which we often entered another stable period of recording.

We used standard spike sorting techniques to isolate single neurons from the measured extracellular potentials (see **Figure S2c-e**, Supplementary Methods S1). We applied conservative criteria to conclude that the neuron isolated on a given electrode was the same as on the previous day. Each day we compared spike waveforms recorded in current session with spike waveforms which that were recorded on previous day. When the spike amplitude, the shape of the action potential, or the shape of the interspike interval time histogram changed, we concluded that the neuron being recorded might not be the same one, and future recordings were from that electrode were no longer considered appropriate for the intersession analysis. Interspike interval histograms from deemed to be stable, single neurons (see **Results**) are shown in **Figure S9**.

### ANOVA analysis and intertrial variability

We analyzed the response of the 69 neurons whose activity was recorded in more than one session. For each cell, we used a 2-way ANOVA to determine the fraction of the response variance that could be accounted by either stimulus selectivity or recording day. First, stimuli were rank ordered based on their spiking response from that cell averaged across all recording sessions, and the 15 stimuli eliciting the highest responses were considered for subsequent ANOVA analysis. Analysis was restricted in this way to in order to minimize the contribution of null responses and focus analysis on the stability in spike rate and temporal patterning. The average spiking histogram (bin size = 20 ms) corresponding to each of these stimuli was then computed for a window between 40 and 440 ms following stimulus appearance. Principal components were extracted from these average histograms providing a set of basis functions used to analyze stability of the response within and between sessions. Spike histograms were then computed for individual trials. The inner product between each of the first six principal components and the individual trial spike histogram was calculated, providing a 6 component score for each trial. The goal of this analysis was to estimate the proportion of variance explained by two factors, recording session and stimulus identity, for either the spike count, or the temporal structure reflected in the principal components.

### Correlation-based similarity index (CSI)

To quantify the relationship between selective spiking responses of individual neurons, we computed an index that took into account both the changes in response magnitude (corresponding to the firing rate selectivity) and the temporal response pattens elecited by each stimulus. This index, termed the correlation-based similarity index (CSI) was computed based on the PSTHs for for all stimuli. Specifically, using a bin width of 40 ms, the PSTH was computed for each stimulus between 40 and 640 ms following stimulus onset. The resulting 16-element vectors for each stimulus were then combined into a single 16×65 martix. The R-values between pairs of such matrices (corresponding to different neurons collected simultaneously, or the same neuron collected over multiple sessions) was then computed using Matlab's corrcoeff function. In the index the degree of similarity ranges from −1.0 to 1.0, with 0.0 corresponding to completely different response pattern, and 1.0 for identical response patterns. The CSI was computed for three different conditions. In the first, response similarity was evaluated between different neurons that were recorded simultaneously from different electrodes. In the second, response similarity was evaluated for different neurons that were recorded simultaneously from the same electrode. In the final condition, the similarity of responses was evaluated for a single neuron over multiple sessions. In all cases, the CSI was computed based upon the responses of a single session containing at least 9 trials. In the case of the intersession similarity, comparisons were made between the first and last session during which the neuron was successfully isolated.

## Supporting Information

Supplementary Methods S1 (0.04 MB DOC)

Figure S1 Visual stimuli used in the present study. Each stimulus subtended an approximately 5 degrees visual angle, and was presented on a CRT computer monitor for either 400 or 500 ms.
(7.06 MB TIF)

Figure S2 Details of neurophysiological recordings. A. Recordings were carried out with bundled microwire electrodes consisting of insulated nickel-chromium wire (12.5 µm diameter cross section). Each wire was individually soldered to a custom-made connector using silver-tin (Castoline 157), allowing for simultaneous recording from all 64 electrodes. B. Example of raw signals obtained simultaneously from 9 electrodes during a recording session. Spikes of different amplitudes can be seen on the different channels (horizontal bar = 1 ms, vertical bar = 100 µV). C. Distribution of signal to noise (SNR) ratios of the raw signal from which isolated units were extracted on each day. Here, SNR is defined as the spike amplitude divided by double the standard deviation of the noise in the raw trace. The black histogram (background) corresponds to all neurons recorded, while the gray histogram corresponds only to those neurons from which visual responses could be elicited. Two example spike waveforms are shown (horizontal bars = 1 ms, vertical bars = 100 µV). D. Cluster analysis of candidate spike waveforms projected onto first and second principal components. In this example, there were three clearly separable units identifiable from a single electrode (horizontal bar = 1 ms, vertical bar = 100 µV). E. Examples of temporal stability of spike waveforms from four neurons collected over multiple sessions (horizontal bars = 1 ms, vertical bars = 100 µV).
(1.85 MB TIF)

Figure S3 Recording apparatus and location. A. Custom-made ball-and-socket bundle implant used in monkey N97. The electrode bundle was attached to the micromanipulator (depicted in green) to permit additional electrode adjustment in the vertical direction. In addition, the ball-and-socket permitted adjustment along a cone sweeping through a broad range of anterior-posterior and medial-lateral positions. B. Structural MRI scans (post-mortem T2-weighted scan for N97 and anesthestized T1-weighted for E98) of the recording positions in the two monkeys. The red arrows show the position of electrodes tips in the brain tissue.
(4.37 MB TIF)

Figure S4 Details in the monitoring of single unit activity over time with the chronic multielectrode bundle during 20 recording sessions in monkey E98 (A) and 53 sessions in monkey N97 (B). The color code corresponds to the number of simultaneously recorded neurons on one electrode. Deep blue corresponds to the absence of spiking activity on the electrodes. Extended periods of deep blue ranging over all channels correspond to absence of recording in particular time period.
(3.76 MB TIF)

Figure S5 Distribution of spike parameters for putative stably isolated neurons (black) and neurons recorded during the same session (grey). A. Distributions were computed between normalized inter-spike time histograms calculated on the basis of neuronal activity recorded form same neuron on two consecutive recording sessions (black) and between different neurons recorded on the same wire (grey). Small values for Euclidian distances demonstrate high degree of similarity between characteristic features of spiking activity. B. Color coding is same as in A. In this case Euclidian distances were calculated between average spike waveforms. Normalized spike waveforms showed higher degree of similarity in case of stably recorded cells.
(0.55 MB TIF)

Figure S6 Complete responses from a single neuron (second neuron in Fig. 1a) to all stimuli over a period of 17 days. Each action potential is depicted by a small point, with different colors corresponding to different days. The vertical white line corresponds to the presentation of the visual stimulus.
(5.58 MB TIF)

Figure S7 Stability of early vs. late phase responses across the population. In each case, the comparison is made between the neuron's response on the first and last recording session. A. Early response, mean spike rate (40–160 ms). B. Late response mean, spike rate (160–440 ms). C. Ratio of early to late spiking responses.
(0.58 MB TIF)

Figure S8 Distribution of duration of stable isolation of recorded cells. Data are shown separately for monkey E98 (black bars) and monkey N97 (gray bars), along with their sum (white bars).
(0.56 MB TIF)

Figure S9 Interspike interval (ISI) histograms for each of 158 neurons recorded in the present study from both monkeys. In one of the neurons (red box), the ISI distribution is polluted by a periodic signal of unknown origin.
(1.53 MB TIF)

Table S1 Details of ANOVA analysis for the neurons shown in Figures 1, 2 and 5.
(0.71 MB TIF)
