# Supplementary material for: Long-Term Stability of Visual Pattern Selective Responses of Monkey Temporal Lobe Neurons
Source: PLoS One. 2009 Dec 9;4(12):e8222. doi: 10.1371/journal.pone.0008222 (PMC2784294; doi:10.1371/journal.pone.0008222)
Supplement: Supplementary Methods S1 — (0.04 MB DOC) [file pone.0008222.s001.doc]

Supplementary Methods

**Surgical procedures.** All surgeries were performed under balanced general anesthesia, using aseptic techniques. Each monkey underwent two surgical procedures. In the first, a head post and scleral eye coil were implanted. Anesthesia was inducted by injection of ketamine-xylazine mixture (ketamine: 10 mg/kg body weight; xylazine: 1-2 mg/kg). A catheter was introduced in the saphenous vein, and the animal was intubated in the trachea and switched to the gas anesthesia. The anesthesia regimen consisted of isoflurane 1.3% and fentanil 3 μg/kg i.v. injections, with 1.8 l/min N_2_O and 0.8 l/min O_2_. Monitoring of anesthesia state was done by the registration of CO_2_ levels, EKG, SpO_2_, temperature and blood pressure. In the first phase of surgery, a scleral search coil was implanted under the scleral conjunctive, and secured with nonabsorbable sutures ^1^. In the second phase, a “U”-shaped skin incision was made and the skin flap reflected. After the muscle was gently separated from the surface of the bone, the head post was secured with titanium screws (Synthes, Switzerland). The eyecoil lead from the first phase was then brought in contact with plugs on the head post. A small cut was then made in the skin flap, which was then fed over the implanted post and resutured at the incision margin.

In the second surgery, following several months of training, the multielectrode bundle was implanted according to coordinates identified in structural MRI scanning. The implants themselves were specifically tailored to the form of the animals’ skulls based on high-resolution (0.5 mm isotropic) scans. The implants and procedures differed in the two monkeys. In the first case (monkey E98) the electrodes were introduced through a 2-mm hole in the cranium through a polyimide guide tube. The guide tube was first stereotaxically positioned so that its end was 10-12 mm from the cortical target point, and then fixed to the skull with bone cement, and then the microelectrode bundle was advanced through it until it reached its predetermined target. Lastly, the microplug was fixed to the skull within a special plastic (polyethyl ether ketone, PEEK) implant. In the second case (monkey N97), we used a titanium ball-and-socket type chamber with a micromanipulator (see **Supp Fig 2**). This construction permitted extensive post-implantation adjustment of the electrode position. During surgery, a small craniotomy was made, and the base of the chamber was first attached to the skull with titanium screws. The microelectrode bundle, protruding through the spherical “ball”, was then slowly lowered into the brain until the ball was seated in the base and the electrodes were 5-7 mm dorsal to the area of interest. The animal was recovered, and the further lowering of the electrodes to their target was done several days later while the animal was awake.

**Recordings and data analysis.** Each day, the protective cap was removed from the connector array, and the animal was plugged into one of two amplifiers (MCP-8, AlphaOmega, Israel; FA32X, Multi Channel Systems GmbH, Germany). All channels were monitored for the presence of isolated neurons, and compared to the yield of the previous days. Channels displaying action potentials were digitized at a rate of 22 kHz and stored onto hard disk. Spike extraction was performed offline by custom made software written with Tcl/Tk (http://www.tcl.tk) and Matlab (MathWorks, Natick, MA). Candidate spikes were first identified by adjusting trigger levels to roughly two times the RMS noise of the signal. The corresponding waveform candidates were then passed to a principal components (PC) based analysis, which provided a set of functions from the data that best described the commonalities in the waveform shape. Individual spike candidates were then projected onto these functions, and the entire population of waveforms could be visualized as a cloud or clouds of points on a two-dimensional plane. Clusters corresponding to isolated spikes were separated from each other and from the noise by manually defining a boundary around clusters of interest. The validity of spikes from the resulting clusters were finally evaluated by verifying that the shortest lags in the spike autocorrelogram was clear. (For a full discussion of this topic, see Refs ^2, 3^). Sorted spikes were then used to construct raster diagrams and peristimulus time histograms. Significance in stimulus-evoked responses was assessed by appling t-Tests, and Kolmogorov-Smirnov tests to the mean spike rates.

**Sparseness and depth of selectivity**. The depth depth of selectivity (DOS) and sparseness indices were computed based on the mean spike rates during the period ranging from 40 to 440 msec after stimulus onset. For each neuron the DOS index was computed according to the following formula^4^,

DOS = [ N – (Σ_i = 1,N_ r_i_ / r_max_) ] / [N – 1],

where r_i_  is the averaged spike rate to the *i*th stimulus in the set of N stimuli, and r_max_ is maximal response in the stimulus set. Depth of selectivity changes from 0 to 1, with the highest DOS values corresponding to neurons responding to only one stimulus.

The sparseness index was computed according to the following formula^5^,

sparseness = (Σ_i = 1,N_ r_i_/N)² / Σ_i = 1,N_ (r_i_²/N) ,

where r_i_  averaged spike rate to the *i*th stimulus in the set of N stimuli. Sparseness ranges from 0 to 1, with 0 indicating that a neuron is highly specialized in its responses, and 1 indicating that neurons

Reference List

1. Judge,S.J., Richmond,B.J., & Chu,F.C. Implantation of magnetic search coils for measurement of eye position: an improved method. *Vision Res.* **20**, 535-538 (1980).

2. Wood,F., Black,M.J., Vargas-Irwin,C., Fellows,M., & Donoghue,J.P. On the variability of manual spike sorting. *IEEE Trans Biomed. Eng* **51**, 912-918 (2004).

3. Lewicki,M.S. A review of methods for spike sorting: the detection and classification of neural action potentials. *Network.* **9**, R53-R78 (1998).

4. Rainer,G. & Miller,E.K. Effects of visual experience on the representation of objects in the prefrontal cortex. *Neuron* **27**, 179-189 (2000).

5. Rolls,E.T. & Tovee,M.J. Sparseness of the neuronal representation of stimuli in the primate temporal visual cortex. *J Neurophysiol.* **73**, 713-726 (1995).
