# Supplementary figures and images for: Long-Term Stability of Visual Pattern Selective Responses of Monkey Temporal Lobe Neurons
Source: PLoS One. 2009 Dec 9;4(12):e8222. doi: 10.1371/journal.pone.0008222 (PMC2784294; doi:10.1371/journal.pone.0008222)

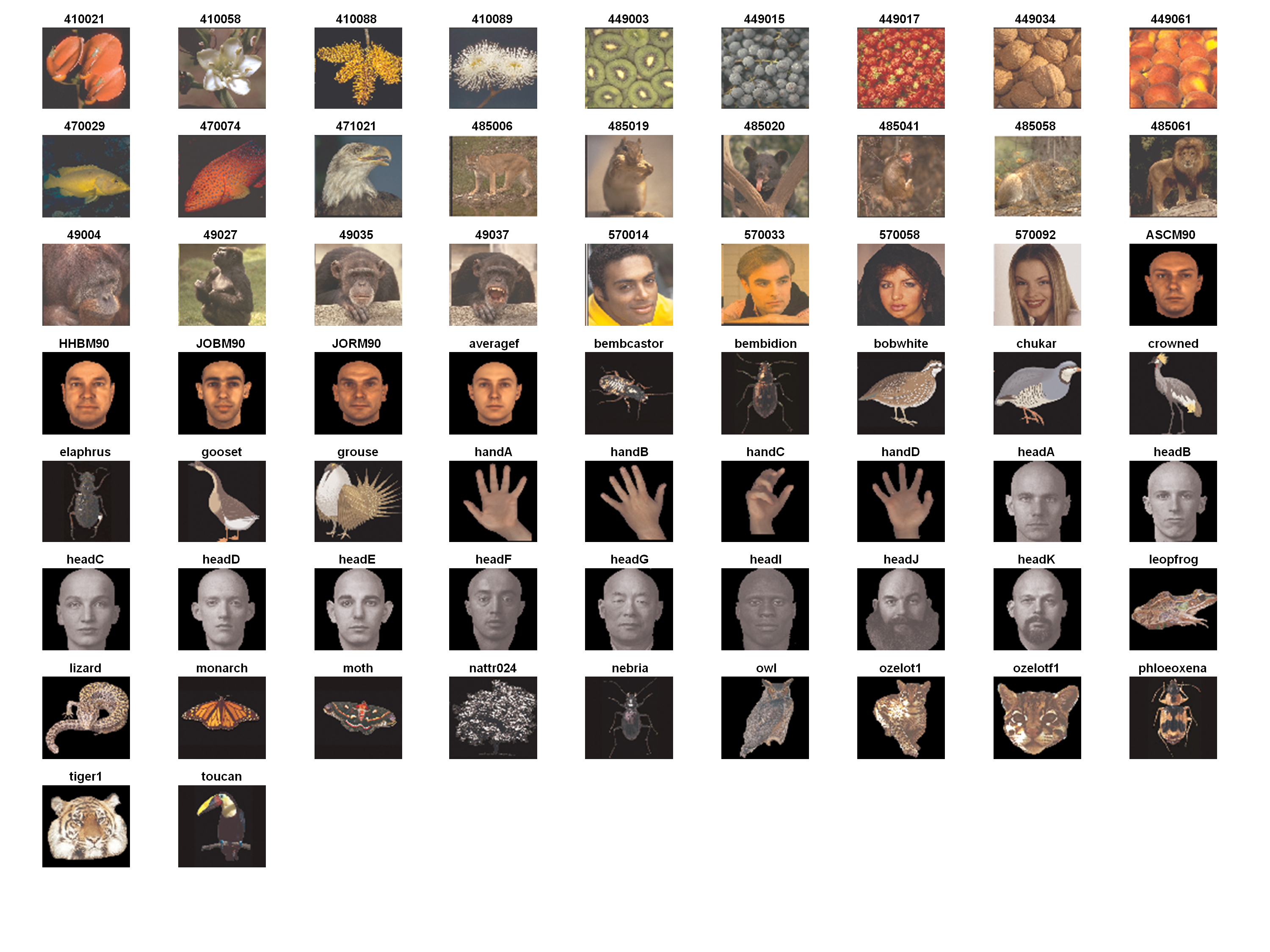

Supplement: Figure S1 — Visual stimuli used in the present study. Each stimulus subtended an approximately 5 degrees visual angle, and was presented on a CRT computer monitor for either 400 or 500 ms. (7.06 MB TIF) [file pone.0008222.s002.tif]

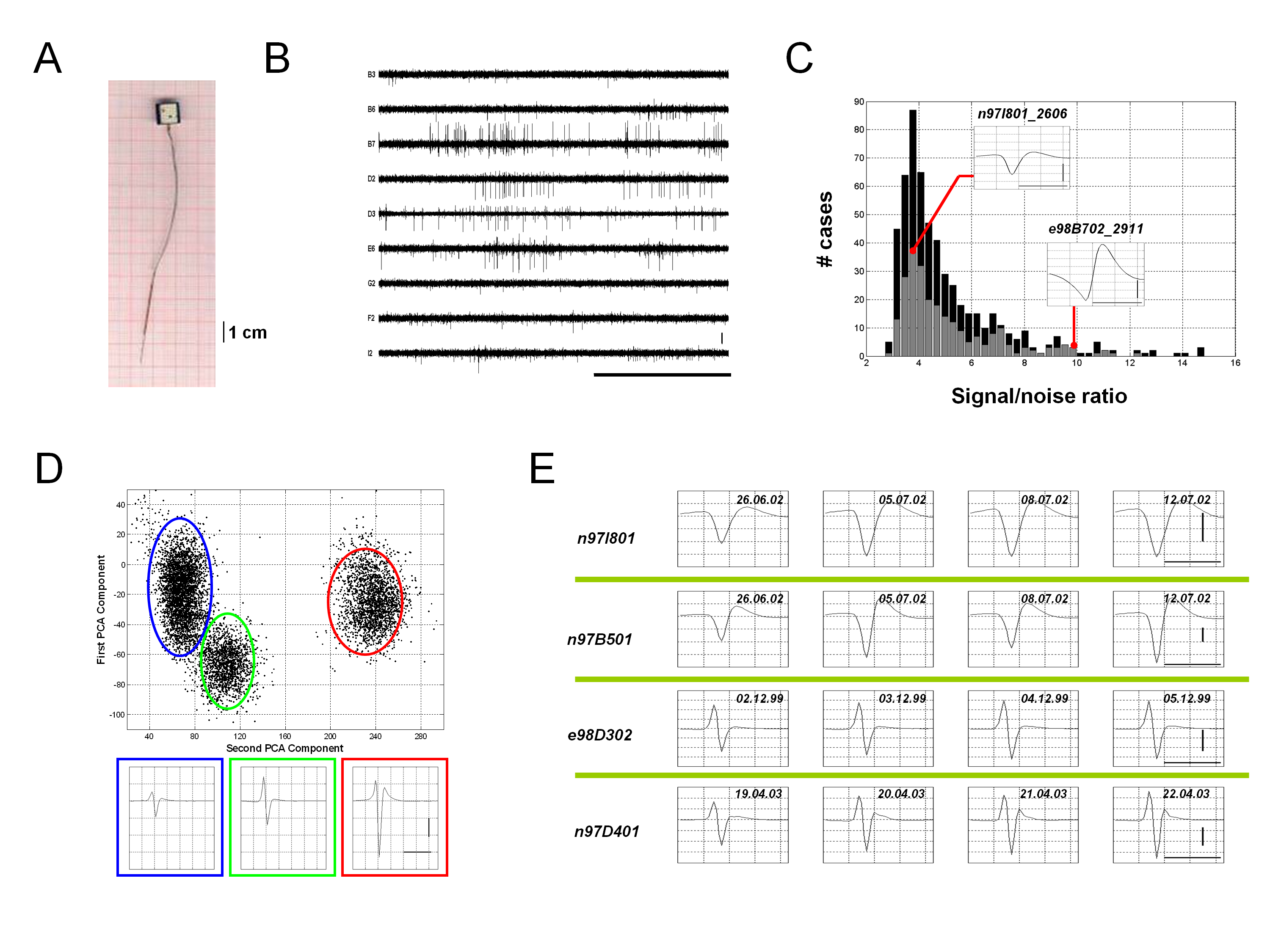

Supplement: Figure S2 — Details of neurophysiological recordings. A. Recordings were carried out with bundled microwire electrodes consisting of insulated nickel-chromium wire (12.5 µm diameter cross section). Each wire was individually soldered to a custom-made connector using silver-tin (Castoline 157), allowing for simultaneous recording from all 64 electrodes. B. Example of raw signals obtained simultaneously from 9 electrodes during a recording session. Spikes of different amplitudes can be seen on the different channels (horizontal bar = 1 ms, vertical bar = 100 µV). C. Distribution of signal to noise (SNR) ratios of the raw signal from which isolated units were extracted on each day. Here, SNR is defined as the spike amplitude divided by double the standard deviation of the noise in the raw trace. The black histogram (background) corresponds to all neurons recorded, while the gray histogram corresponds only to those neurons from which visual responses could be elicited. Two example spike waveforms are shown (horizontal bars = 1 ms, vertical bars = 100 µV). D. Cluster analysis of candidate spike waveforms projected onto first and second principal components. In this example, there were three clearly separable units identifiable from a single electrode (horizontal bar = 1 ms, vertical bar = 100 µV). E. Examples of temporal stability of spike waveforms from four neurons collected over multiple sessions (horizontal bars = 1 ms, vertical bars = 100 µV). (1.85 MB TIF) [file pone.0008222.s003.tif]

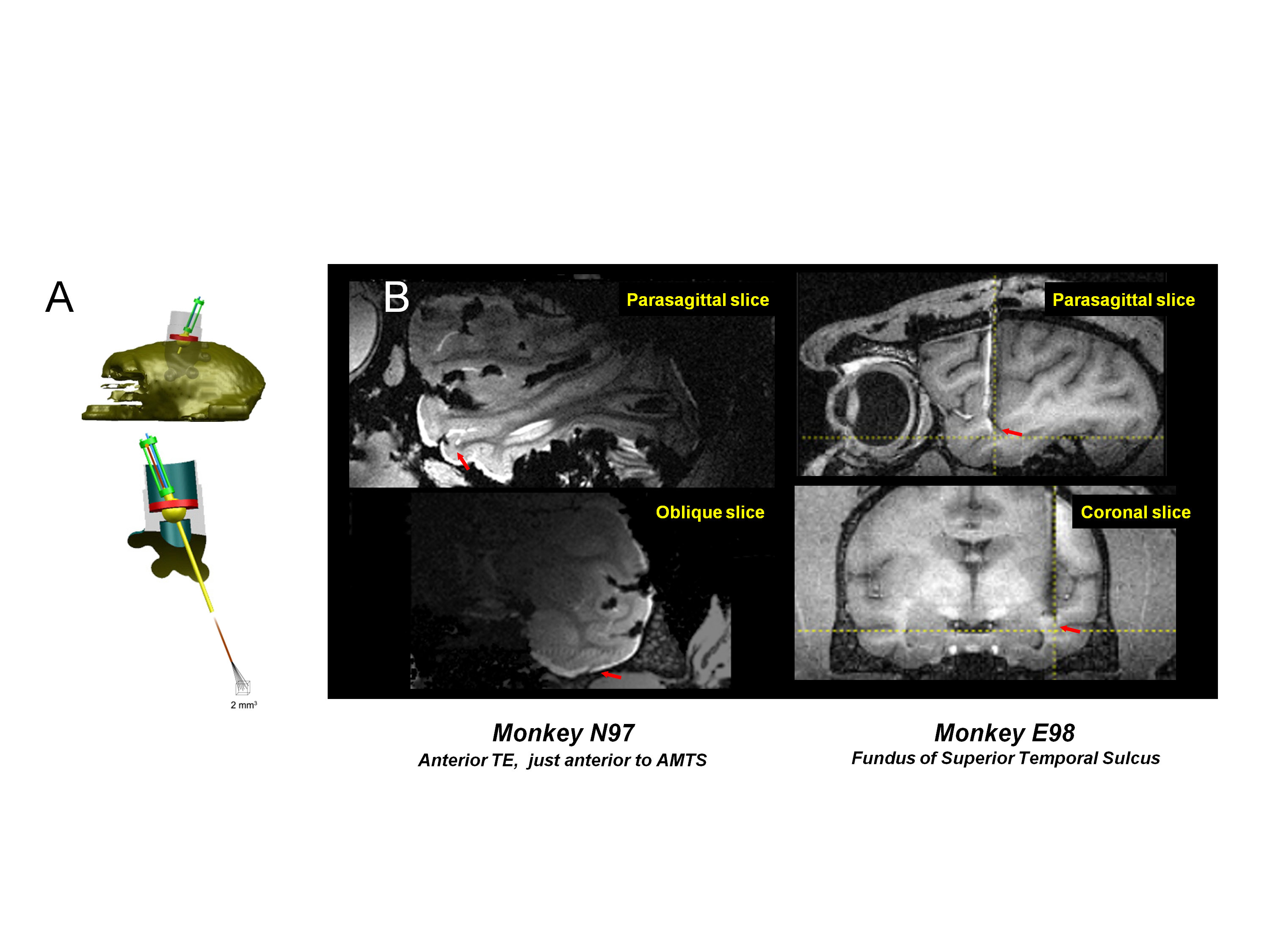

Supplement: Figure S3 — Recording apparatus and location. A. Custom-made ball-and-socket bundle implant used in monkey N97. The electrode bundle was attached to the micromanipulator (depicted in green) to permit additional electrode adjustment in the vertical direction. In addition, the ball-and-socket permitted adjustment along a cone sweeping through a broad range of anterior-posterior and medial-lateral positions. B. Structural MRI scans (post-mortem T2-weighted scan for N97 and anesthestized T1-weighted for E98) of the recording positions in the two monkeys. The red arrows show the position of electrodes tips in the brain tissue. (4.37 MB TIF) [file pone.0008222.s004.tif]

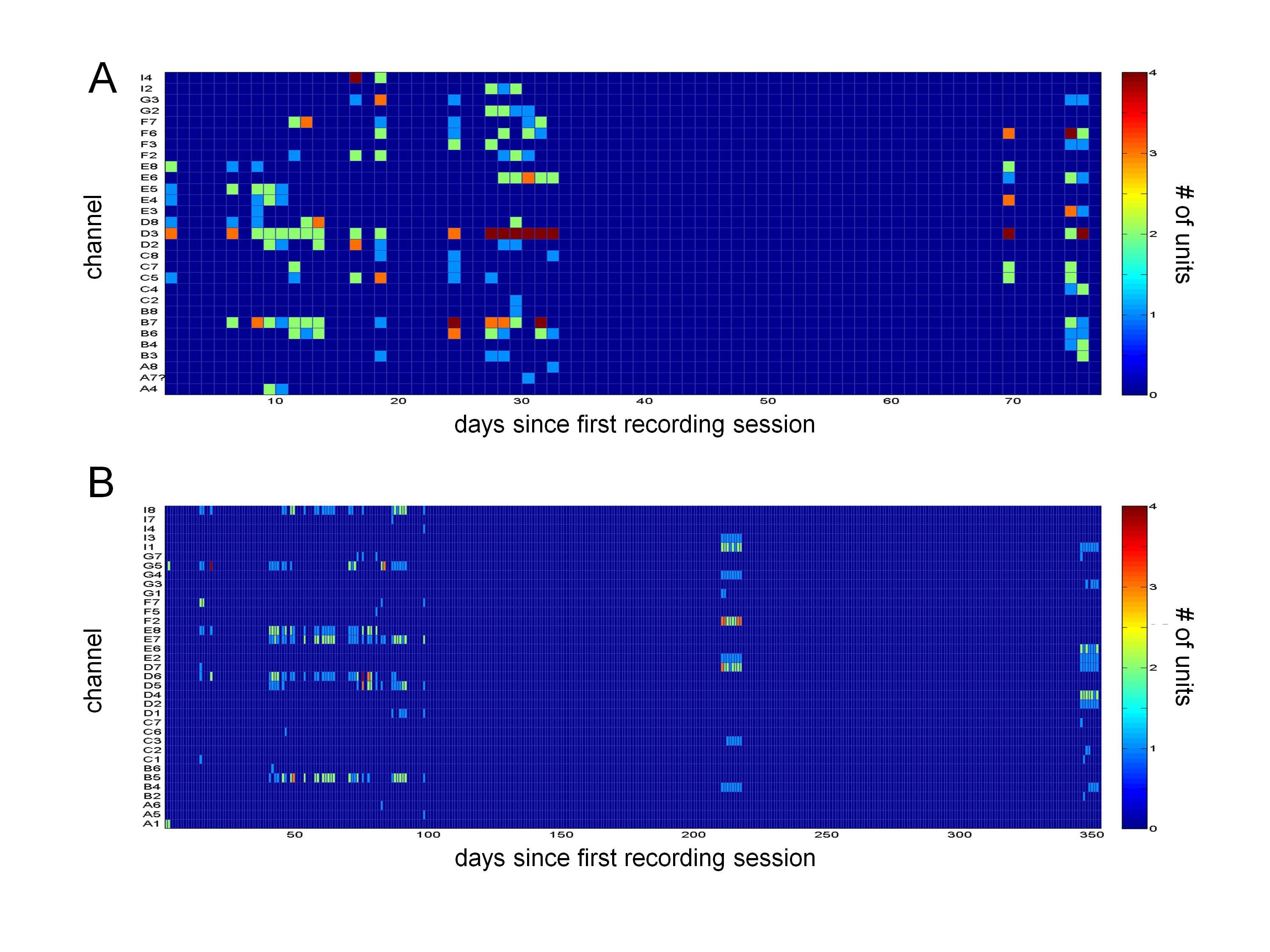

Supplement: Figure S4 — Details in the monitoring of single unit activity over time with the chronic multielectrode bundle during 20 recording sessions in monkey E98 (A) and 53 sessions in monkey N97 (B). The color code corresponds to the number of simultaneously recorded neurons on one electrode. Deep blue corresponds to the absence of spiking activity on the electrodes. Extended periods of deep blue ranging over all channels correspond to absence of recording in particular time period. (3.76 MB TIF) [file pone.0008222.s005.tif]

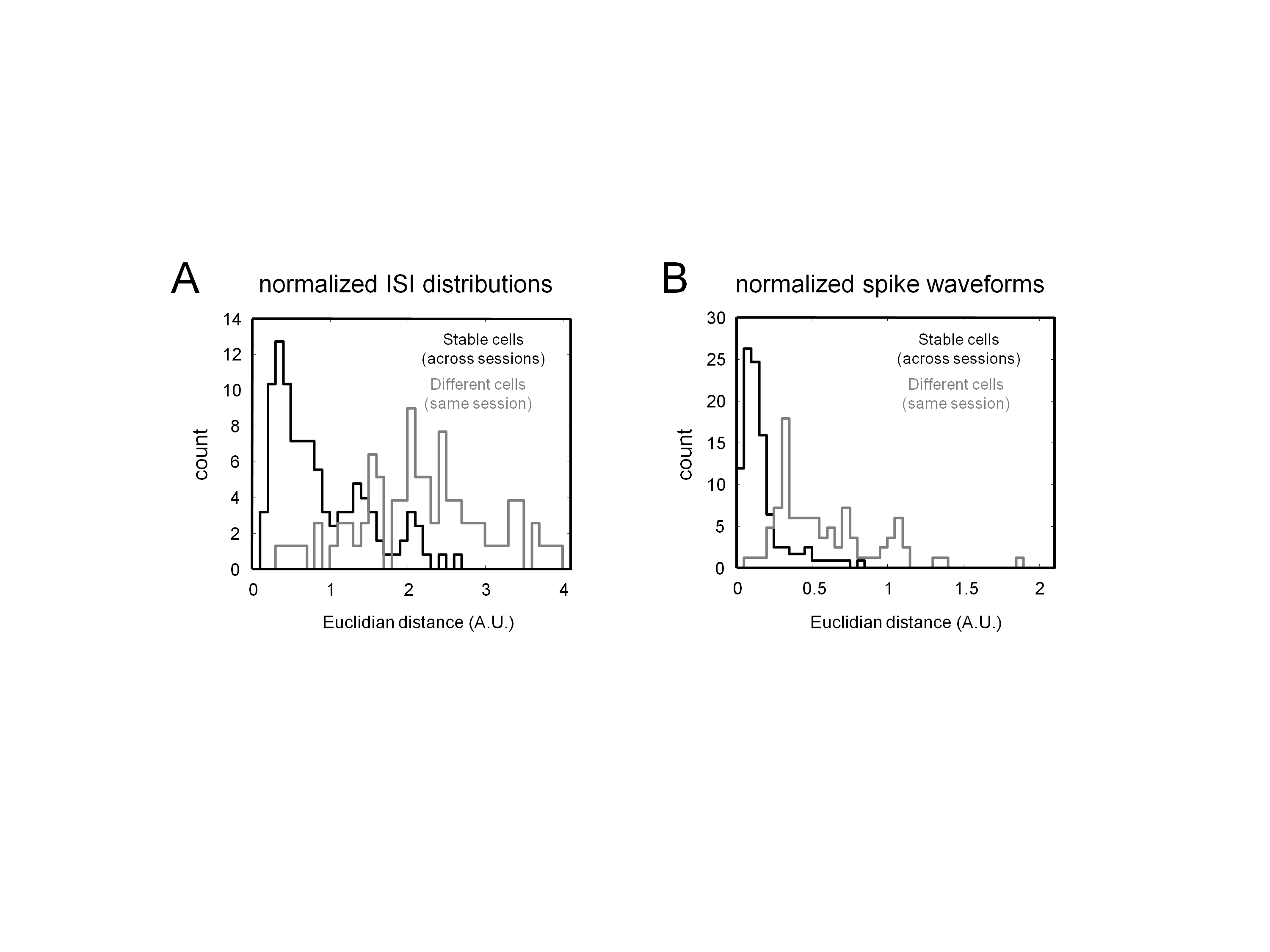

Supplement: Figure S5 — Distribution of spike parameters for putative stably isolated neurons (black) and neurons recorded during the same session (grey). A. Distributions were computed between normalized inter-spike time histograms calculated on the basis of neuronal activity recorded form same neuron on two consecutive recording sessions (black) and between different neurons recorded on the same wire (grey). Small values for Euclidian distances demonstrate high degree of similarity between characteristic features of spiking activity. B. Color coding is same as in A. In this case Euclidian distances were calculated between average spike waveforms. Normalized spike waveforms showed higher degree of similarity in case of stably recorded cells. (0.55 MB TIF) [file pone.0008222.s006.tif]

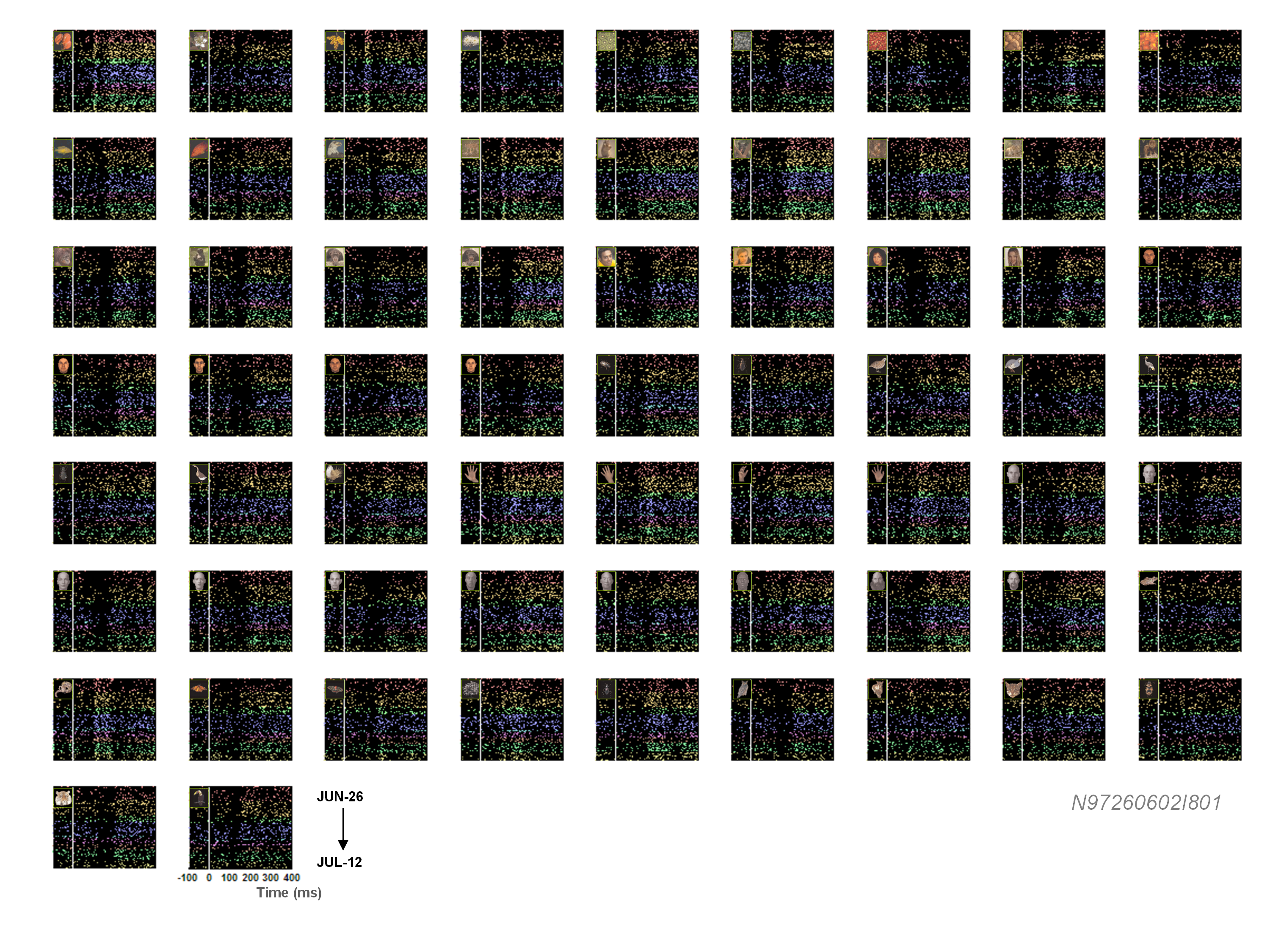

Supplement: Figure S6 — Complete responses from a single neuron (second neuron in Fig. 1a) to all stimuli over a period of 17 days. Each action potential is depicted by a small point, with different colors corresponding to different days. The vertical white line corresponds to the presentation of the visual stimulus. (5.58 MB TIF) [file pone.0008222.s007.tif]

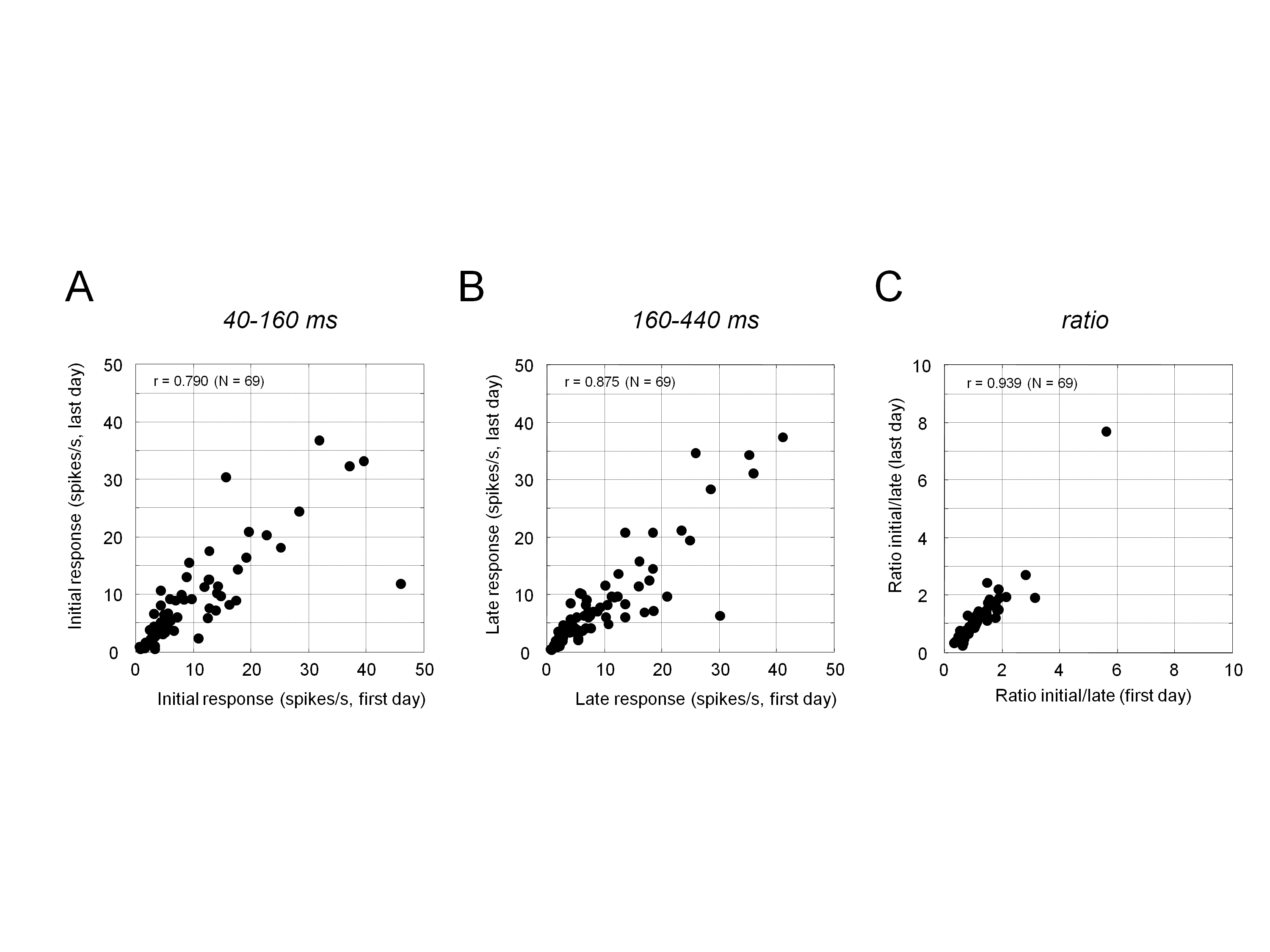

Supplement: Figure S7 — Stability of early vs. late phase responses across the population. In each case, the comparison is made between the neuron's response on the first and last recording session. A. Early response, mean spike rate (40–160 ms). B. Late response mean, spike rate (160–440 ms). C. Ratio of early to late spiking responses. (0.58 MB TIF) [file pone.0008222.s008.tif]

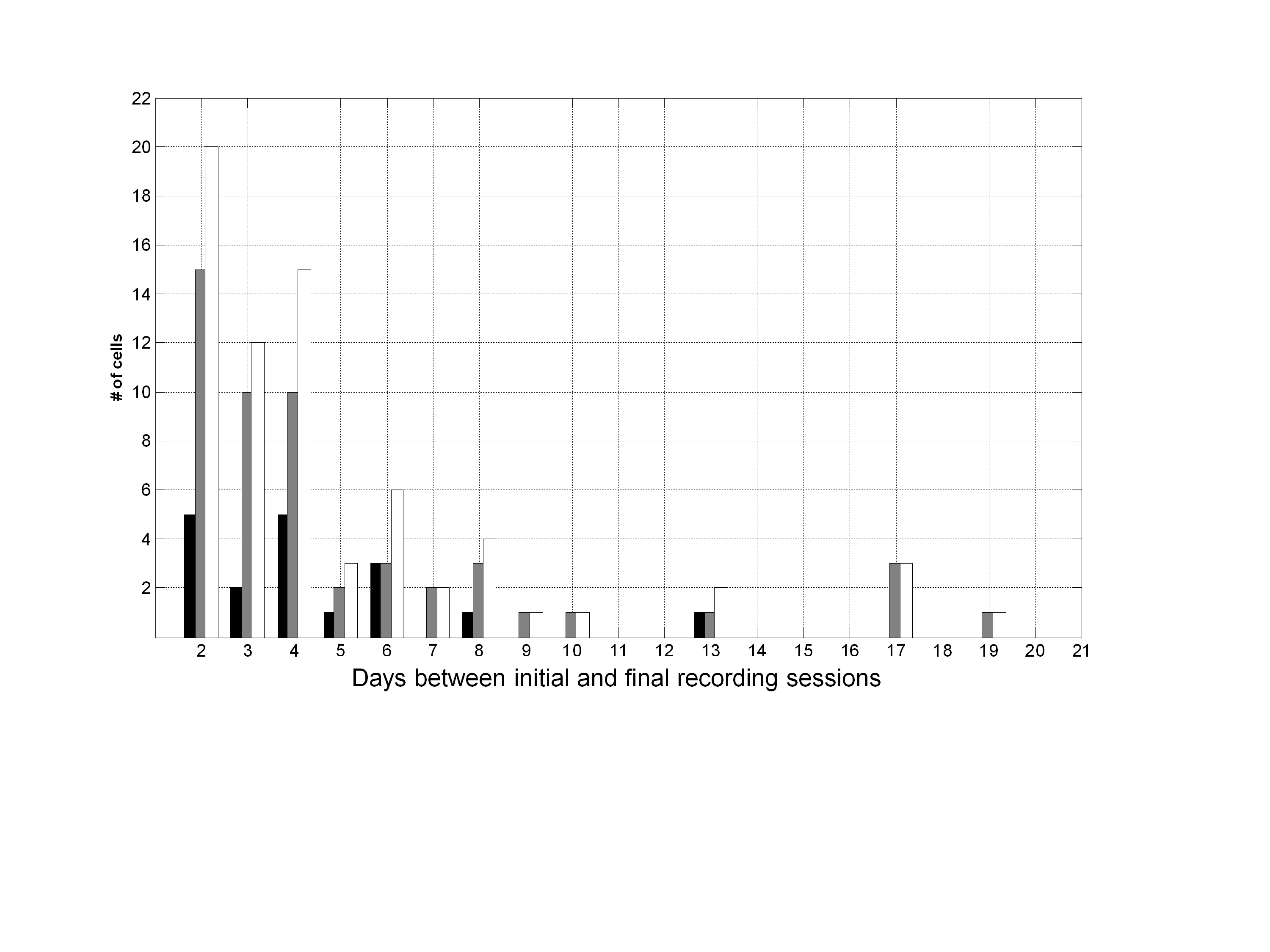

Supplement: Figure S8 — Distribution of duration of stable isolation of recorded cells. Data are shown separately for monkey E98 (black bars) and monkey N97 (gray bars), along with their sum (white bars). (0.56 MB TIF) [file pone.0008222.s009.tif]

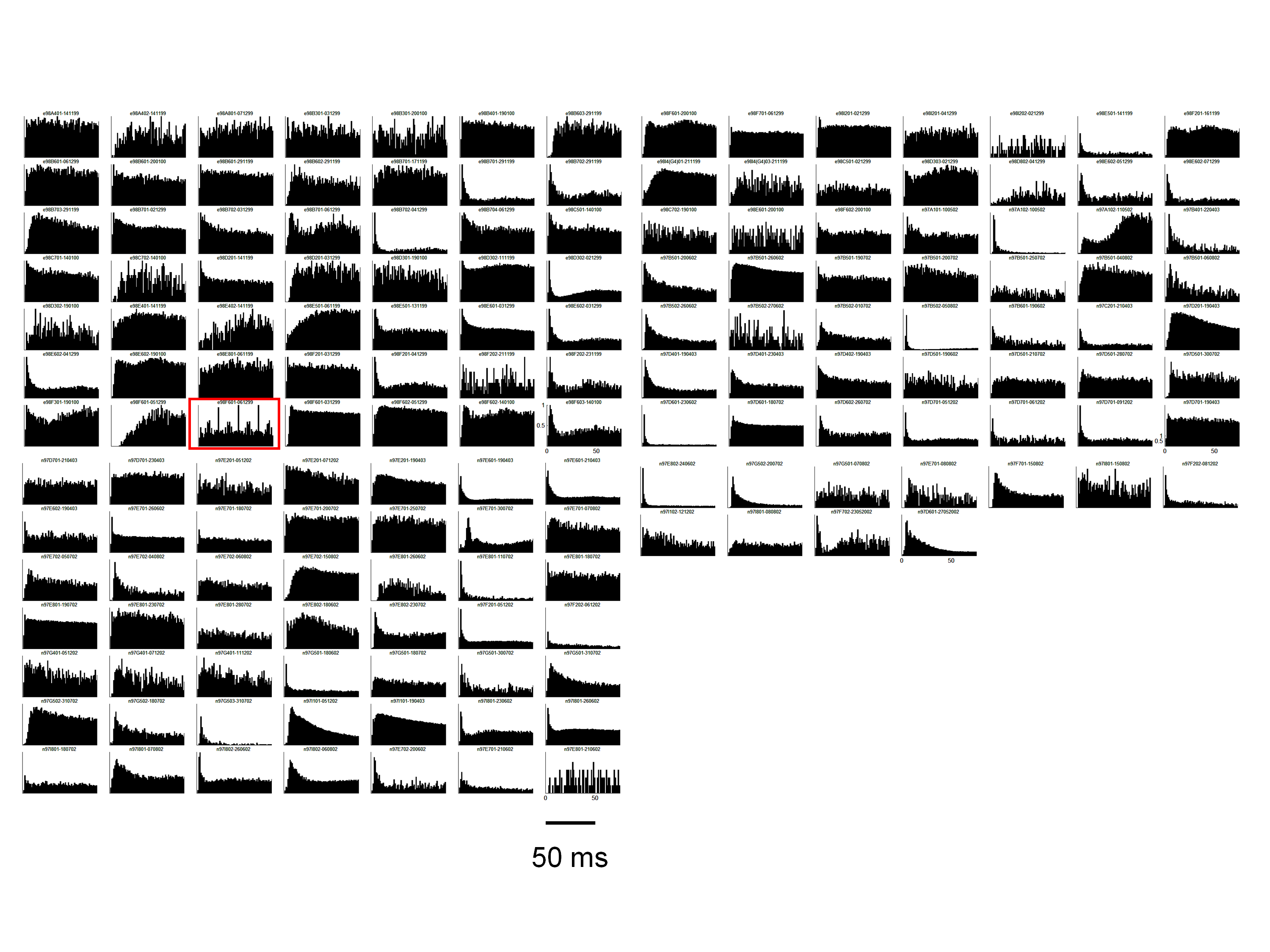

Supplement: Figure S9 — Interspike interval (ISI) histograms for each of 158 neurons recorded in the present study from both monkeys. In one of the neurons (red box), the ISI distribution is polluted by a periodic signal of unknown origin. (1.53 MB TIF) [file pone.0008222.s010.tif]

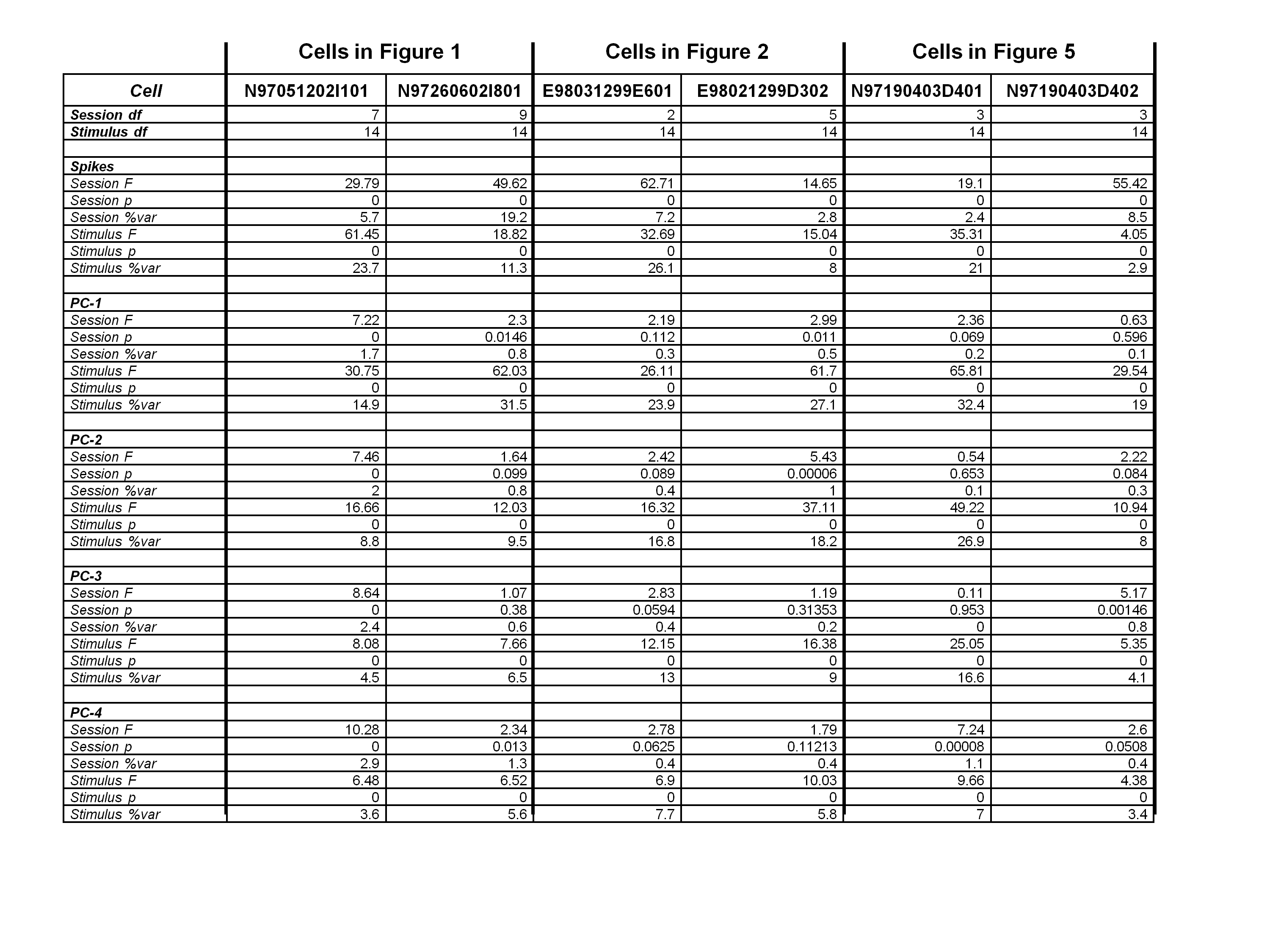

Supplement: Table S1 — Details of ANOVA analysis for the neurons shown in Figures 1, 2 and 5. (0.71 MB TIF) [file pone.0008222.s011.tif]
